# The efficacy of a brief app-based mindfulness intervention on psychosocial outcomes in healthy adults: A pilot randomised controlled trial

**DOI:** 10.1371/journal.pone.0209482

**Published:** 2018-12-31

**Authors:** Louise Champion, Marcos Economides, Chris Chandler

**Affiliations:** 1 School of Social Sciences, London Metropolitan University, London, United Kingdom; 2 Headspace Inc., Los Angeles, California, United States of America; Brown University, UNITED STATES

## Abstract

**Background:**

Previous evidence suggests that mindfulness training may improve aspects of psychosocial well-being. Whilst mindfulness is traditionally taught in person, consumers are increasingly turning to mindfulness-based smartphone apps as an alternative delivery medium for training. Despite this growing trend, few studies have explored whether mindfulness delivered via a smartphone app can enhance psychosocial well-being within the general public.

**Methods:**

The present pilot randomised controlled trial compared the impact of engaging with the self-guided mindfulness meditation (MM) app ‘Headspace’ (n = 38) for a period of 10 or 30 days, to a wait-list (WL) control (n = 36), using a cohort of adults from the general population. The Satisfaction with Life Scale, Perceived Stress Scale, and Wagnild Resilience Scale were administered online at baseline and after 10 and 30 days of the intervention.

**Results:**

Twelve participants (MM n = 9, WL n = 3) were lost to follow-up for unknown reasons. Relative to the WL control, the MM app positively impacted self-reported satisfaction with life, stress, and resilience at day 10, with further improvements emerging at day 30 (Cohen’s *d* = 0.57, 1.42, 0.63 respectively). The rate of improvement was largest at the 10-day assessment point, dropping moderately by day 30. Participants that rated the MM app as easy to engage with experienced the largest self-reported benefits. Moreover, the MM app was able to protect against an unexpected increase in perceived stress that emerged in the control group.

**Conclusions:**

This pilot randomised controlled trial shows that self-reported improvements in psychosocial outcomes can be achieved at low cost through short-term engagement with a mindfulness-based smartphone app, and should be followed up with more substantive studies.

**Trial registration:**

ISRCTN ISRCTN34618894.

## Introduction

Mindfulness can be defined as attending to one’s present-moment thoughts, feelings and sensations, with an open and curios mind, and without attempting to change the experience [1]. The practice of mindfulness meditation (MM) dates back several thousand years, though it was not until the 1970s that it became popular in the West, with Jon Kabat-Zinn introducing Mindfulness-Based Stress Reduction (MBSR; [2]) as an intervention for individuals with stress-related disorders. Early empirical investigation into the effects of MM on physical and mental health suggested that mindfulness increases attentiveness and awareness of present-moment experience [3,4], which in turn is associated with greater psychological well-being [5,6].

MBSR [2] and Mindfulness-Based Cognitive Therapy (MBCT; a variant of MBSR designed as a relapse-prevention treatment for depression [7]) are two of the most prominent and well-researched mindfulness-based interventions to date. Recent meta-analyses have shown that these interventions are effective at reducing stress, and symptoms of depression and anxiety in both healthy and clinical populations [8,9]. Moreover, mindfulness-based interventions have been shown to reduce occupational stress and burnout in teachers [10], doctors [11] and military personnel [12]. Researchers have also identified a positive relationship between trait mindfulness and resilience, with greater resilience being associated with higher levels of psychosocial well-being [13].

Recently, there is growing interest in digital and online tools as an alternative delivery medium for health and well-being interventions [14]. In particular, smartphone apps have the potential to offer a broad range of content that is both interactive and dynamic, whilst also being cost-effective, flexible, and widely accessible. Such attributes support the promotion of using smartphones in self-guided well-being interventions [15], and this is reflected in a recent surge in the number of individuals utilising commercially-available mindfulness-based smartphone apps.

Despite this growing trend, few empirical studies have investigated whether commercial MM apps are effective at improving aspects of phycological well-being. Recent meta-analyses suggest that online MM training confers similar benefits to in-person MBSR training with respect to stress, anxiety, and depression, albeit with more modest effect sizes for the latter two outcomes [8,16]. However, online MM programs may substantially differ from commercial MM apps across important attributes such as session length, frequency of practice, program structure, and the degree of user interactivity. Together this suggests that there is substantial need for studies that specifically investigate the impact of commercial MM apps, many of which are now being used extensively by the general public.

The popularity of mindfulness training in healthy populations may reflect a desire for people to develop strategies to cope with the strains of daily life, and to build resilience against developing poor mental health in the future. Yet, few studies have investigated whether MM apps can enhance aspects of psychosocial well-being, or increase resilience [17,18]. One randomised controlled trial (RCT) found that ten sessions of the MM app ‘Headspace’ led to improvements in self-reported stress, affect and irritability [19], whilst another found increases in self-reported quality of life and reductions in general psychiatric symptoms following an 8-week intervention using the app ‘VGZ Mindfulness Coach’ [20]. A recent study by Howells and colleagues, which also utilised ten sessions of Headspace, found increases in self-reported positive affect and reductions in depressive symptoms in a cohort of self-identifying ‘happiness seekers’, but no increase in satisfaction with life [21]. Happiness seekers are those who intentionally invest in their own well-being through happiness-promoting strategies, and who experience above average symptoms of depression relative to the general population [22]. Howells and colleagues speculated that self-reported satisfaction with life may have increased if their app-based MM intervention has been longer.

The present pilot RCT aimed to replicate and extend Howells and colleagues’ study by including a longer intervention period, a study cohort more representative of the general population, and new self-reported outcome measures. Specifically, the intervention period was extended to 30 days, encompassing the app’s full introductory program, with outcomes being compared to a wait-list control at days 10 and 30 of the intervention. The introductory program features daily MMs (of approximately 10 to 20 minutes in duration) guided by former Buddhist monk Andy Puddicombe. By utilising a longer intervention, the present study was able to test Howells and colleagues’ hypothesis that satisfaction with life would increase following a greater volume of MM, as well as differentiate between the impact of short and medium periods of MM on self-reported well-being outcomes. Perceived stress and resilience were included as outcome measures, as very few studies of app-based MM have studied these outcomes, despite them being key indicators of long-term psychological well-being [13,23]. Finally, by utilising a community cohort of health adults, the present study aimed to investigate whether Howells and colleagues’ findings in individuals that demonstrate heightened levels of depression would extend to a study sample more representative of the general population. This is important because commercial MM apps are targeted at the general public and emphasize the importance of mindfulness as a tool for increasing general health and happiness.

It was hypothesized that relative to controls, the MM group would experience significant beneficial impact with regards to self-reported satisfaction with life, perceived stress, and resilience, and that the extent of this benefit (particularly for satisfaction with life), would be greater following 30 days of the intervention than 10 days. It was also hypothesized that users with higher self-rated task enjoyment (or lower self-rated task difficulty) would experience the largest positive change across all three outcomes, as this was previously demonstrated by Howells and colleagues. Previous theoretical [24] and empirical [25] evidence also suggests that intervention enjoyment can moderate the degree to which participants engage with and benefit from wellness interventions [26].

## Materials and methods

### Participants

Participants were recruited via opportunity and snowball sampling. A mass email (see appendix of ‘S1 Protocol’) was sent to a cohort of employees belonging to the same organisation inviting volunteers from the general population to take part in a study on mindfulness and well-being (note that employees were targeted for convenience only, and the study did not aim to investigate workplace wellness). As this was a pilot study, there was no a priori planned sample size. However, following recent guidelines for pilot RCTs we aimed to include approximately 70 participants [27]. The email was sent to approximately 260 individuals, 105 of which expressed interest, with 13 being immediately excluded due to having at least 20 minutes of prior meditation experience. 92 participants (54 females and 38 males), with ages ranging from 25–59 years, proceeded to complete a questionnaire which included demographics and a group of items used to screen for inclusion / exclusion criteria (see ‘Screening tool’ under Measures). Exclusion criteria were set based on guidelines from MBSR [28] and MBCT [29], and included i) engagement with mindfulness or meditation for more than 20 total minutes, ii) a history of, presence, or ongoing treatment for a psychological disorder, iii) a score greater than 11 on the General Health Questionnaire (GHQ) or a positive answer to questions 27 and 28 regarding suicidality. Inclusion criteria included being at least 18 years of age, and having access to a smartphone.

Of the 92 participants screened, 74 were deemed eligible (41 female, 33 male), with ages ranging from 25–59 years (mean = 39.4, SD = 5.76). The sample size was thus in keeping with numbers recommended for pilot RCTs [27]. The majority of the participants were based in the UK (n = 72) with just two participants located in the U.S. Since the study was conducted entirely online, all contact with participants was via email. Participant recruitment began in April 2016 and data collection ended in August 2016. The study was approved by the London Metropolitan University Ethics Committee (13050652), and all procedures performed were in accordance with the ethical standards of the London Metropolitan University and with the 1964 Helsinki declaration and its later amendments or comparable ethical standards. Written informed consent was obtained from all individual participants included in the study. The study is registered with the ISRCTN registry (ISRCTN34618894). Due to the pilot nature of this work, the authors were unaware that the study formally met the WHO definition of a clinical trial, and thus registration was done retrospectively. The authors confirm that all ongoing and related trials for this intervention are registered.

### Procedure

The study employed a repeated-measures, randomized controlled design. Participants were emailed a screening questionnaire via Survey Monkey (www.surveymonkey.com) to assess inclusion/exclusion criteria. Those that that did not meet the criteria were informed via email and thanked for their time. The remaining participants were randomised (using simple randomization) via a computer-generated sequence with equal weight to the MM group or WL control group. Sequence generation and randomization was performed by the research team, who were not formally blinded to group allocation. Participants were then emailed a link to the baseline questionnaires (see Measures below), which they were instructed to complete immediately, and provided with a description of what to expect from the study over the following 30 days (see appendix of ‘S1 Protocol’ for briefing email). Those randomised to the MM group (n = 38) were also sent a code which provided 30-days of free access to the MM app, and instructions on how to download the app and redeem their code. Participants were encouraged to begin the app’s introductory program within 24 hours of receiving their code. The program encourages users to self-administer 10–20 minutes of MM daily for a total of 30 days. Participants were emailed the study questionnaires again following 10 days, which included a set of user experience questions and a note encouraging them to continue using the app each day until the third and final set of questionnaires, which was sent to participants on day 30. Participants that did not complete the final set of questionnaires were sent up to three reminder emails, after which they were considered to have withdrawn.

The WL group were informed that they would not be engaging with any MM content until day 30 of the study (see appendix of ‘S1 Protocol’ for briefing email). As with the MM group, they were instructed to complete follow-up questionnaires at days 10 and 30, and received up to three follow-up emails if they failed to do so. Participants were informed that on completion of the final questionnaires they would receive a code providing 30 days of free access to the MM app. Neither group of participants were given any additional compensation for participating.

### Mindfulness intervention

Headspace consists of guided MMs delivered by former Buddhist monk Andy Puddicombe, and is available on Apple iOS or Android devices (including tablets), as well as via the company’s website. Participants were instructed to complete the app’s introductory program which consists of three levels, named “Foundations 1–3”, with each level comprising 10 sessions (30 in total). The program is intended to introduce the key principles behind mindfulness, and how one can apply mindfulness to their daily life, using technique such as breath awareness, body scanning, and noting. Sessions begin with a duration of 10 minutes, though users have the option to increase the duration to 15 and 20 minutes during levels 2 and 3 respectively. The audio content is supplemented with educational videos and animations. At the time of writing, the app has been downloaded more than 30 million times, was recently ranked the highest scoring mindfulness-based iPhone app as per the Mobile Application Rating System [18], and has previously been shown to increase compassion [30], self-reported well-being [21], and self-reported mindfulness [31–33], and to reduce mind-wandering, self-reported stress, and self-reported irritability [19,33].

### Measures

#### Screening tool

As previously mentioned, a screening tool was used which captured the following information: i) demographics (age & gender), ii) mindfulness and meditation experience, iii) history of psychological problems, iv) treatment for psychological problems, v) experience of recent life events such as bereavement, vi) whether they expected difficult future events, and vii) a list of psychological illnesses including suicidality, physical addictions and post-traumatic stress disorder. The General Health Questionnaire (GHQ; [34]) was also included following recent recommendations [35].

#### General Health Questionnaire 28

The GHQ [34] is a 28-item measure specifically designed as a screening tool and to assess four factors of distress—depression, anxiety, social impairment and hypochondriasis. The questionnaire includes statements such as “Have recently lost much sleep over worry”, to which participants must respond by taking into account the previous two weeks. For each of the 28 items, a response of “not at all” or “no more than usual” is assigned a score of 0, whilst a response of “more than usual” or “much more than usual” is assigned a score of 1. Scores are then summed, with a higher total score indicating greater distress (min = 0, max = 28). Test-retest reliability for the GHQ has been reported to be high (0.78 to 0.9) and interrater and inter-rater reliability have both been shown to be excellent (Cronbach's α 0.9–0.95) [36].

#### Satisfaction with Life Scale

The Satisfaction with Life Scale (SWLS; [37]) is a 5-item scale that assesses global satisfaction (e.g. ‘so far I have gotten the important things I want in my life’). Individuals indicate their degree of agreement or disagreement with the items using a 7-point Likert scale (1 = strongly disagree to 7 = strongly agree). The scale is shown to have strong psychometric properties [38] including internal consistency (α = .87) and a high test-retest reliability (α = .82; [39]).

#### Perceived Stress Scale

The Perceived Stress Scale (PSS; [40]) measures the degree to which situations in an individual’s life might be interpreted as stressful. The 10-item scale aims to establish how unpredictable, uncontrollable and overloaded respondents’ find their lives with respect to the previous month. Respondents answer questions about their feelings and thoughts using a 5-point Likert scale (0 = never to 4 = very often). Scores range from 0–40 with higher scores indicating more stress. The scale has high test-retest reliability (α = .85) and its validity with other measures ranges from .52-.76.

#### Wagnild Resilience Scale

The Wagnild Resilience Scale (WRS; [41]) is a 14-item measure which focuses on positive psychological qualities as opposed to deficits. The items reflect five positive characteristics of resilience; self-reliance, purpose, equanimity, perseverance and existential aloneness (see [41] for a full explanation of each characteristic). Respondents answer questions using a 7-point Likert scale (1 = disagree to 7 = agree). Scores can range from 14–98, with higher total scores indicating more resilience. The WRS has good construct validity and internal reliability (α = .94).

#### Engagement and experience questionnaire

An engagement and experience questionnaire was delivered to the MM group at the 10 and 30-day assessment points. Based on Howell et al. (2014), respondents were also asked to rate their experience of using the app. Participants were asked to select the number of days they had used the application (from 1–10 between baseline and day 10, and from 1–20 between days 11 and 30), as well as the duration of each session between sessions 11–30 (10, 15, or 20 minutes). Participants were also asked two further engagement-related questions: 1) “To what to extent are you finding completing the activities enjoyable?” (1 = not at all, to 7 = extremely), and 2) “To what extent are you finding the activity difficult to complete?” (1 = very difficult, to 7 = very easy).

### Statistical analyses

The present study included both an intention-to-treat (ITT) and complete-case analysis. For the former, outcome scores at baseline, day 10, and day 30 were analysed using linear mixed effects models, as these have the ability to handle missing data and are considered to be superior to other ITT approaches such as ‘last observation carried forward’ [42,43]. A number of nested models were fit using maximum likelihood and compared using likelihood ratio tests. For all outcomes, the winning model included time (coded as 0, 10 & 30), group (coded as 1 & 0) and their interaction as fixed effects variables, and random intercepts and slopes across participants. Covariance between the random intercept and slope was modelled for outcomes in which this improved the model fit.

For the complete case analysis, participants that completed the outcome measures at all three assessment points were included, regardless of their self-reported meditation activity (i.e. even participants with very minimal app engagement were included). To assess the impact of the MM app on outcome measures, a 2 (group) x 3 (time) analysis of variance (ANOVA) was implemented for each dependent variable (SWLS, PSS, WRS). Post-hoc paired t-tests were used to interrogate any main effects or interactions. Within-group Cohen’s d effect sizes were calculated with (M_post_ − M_pre_)/SD_pooled_. Between-group Cohen’s d effect sizes were calculated from the difference in score changes between the study conditions divided by the pooled standard deviation at baseline [44]. In order to rule out the possibility that attrition could have influenced our estimates of Cohen’s d, we used multiple imputation (MI) to impute missing values and re-calculated Cohen’s d using the full imputed dataset [45]. The MI procedure used the data augmentation method [46], with default values of 10 independent chains of length 100. 95% confidence intervals (CIs) were calculated for all Cohen’s d values using equations 15 and 18 (for within-group effect sizes) and equations 15 and 16 (for between-group effect sizes) from Nakagawa and Cuthill, 2007 [47].

Exploratory multiple regressions were performed to investigate the influence of demographics, self-rated intervention enjoyment / level of difficulty, and intervention engagement on change in outcome scores from pre to post intervention (in the MM group only). In each case, change in outcome score (in SWLS, PSS, or WRS) from baseline to day 10, or day 10 to day 30, was the dependent variable. Explanatory variables included age, gender (coded as male = 0, female = 1), and self-rated task difficulty (1 = very difficult, to 7 = very easy). Engagement (total number of sessions completed) and self-rated task enjoyment were not included in the final regressions, as these variables were highly correlated with self-rated difficulty (resulting in high levels of multicolinearity). Self-rated task difficulty was chosen as it captured the highest proportion of shared variance amongst the three variables. All statistical analyses were completed using Matlab R2016b (www.mathworks.com) and IBM SPSS Statistics 22, and computed at p < 0.05.

## Results

### Attrition

**Fig 1:**
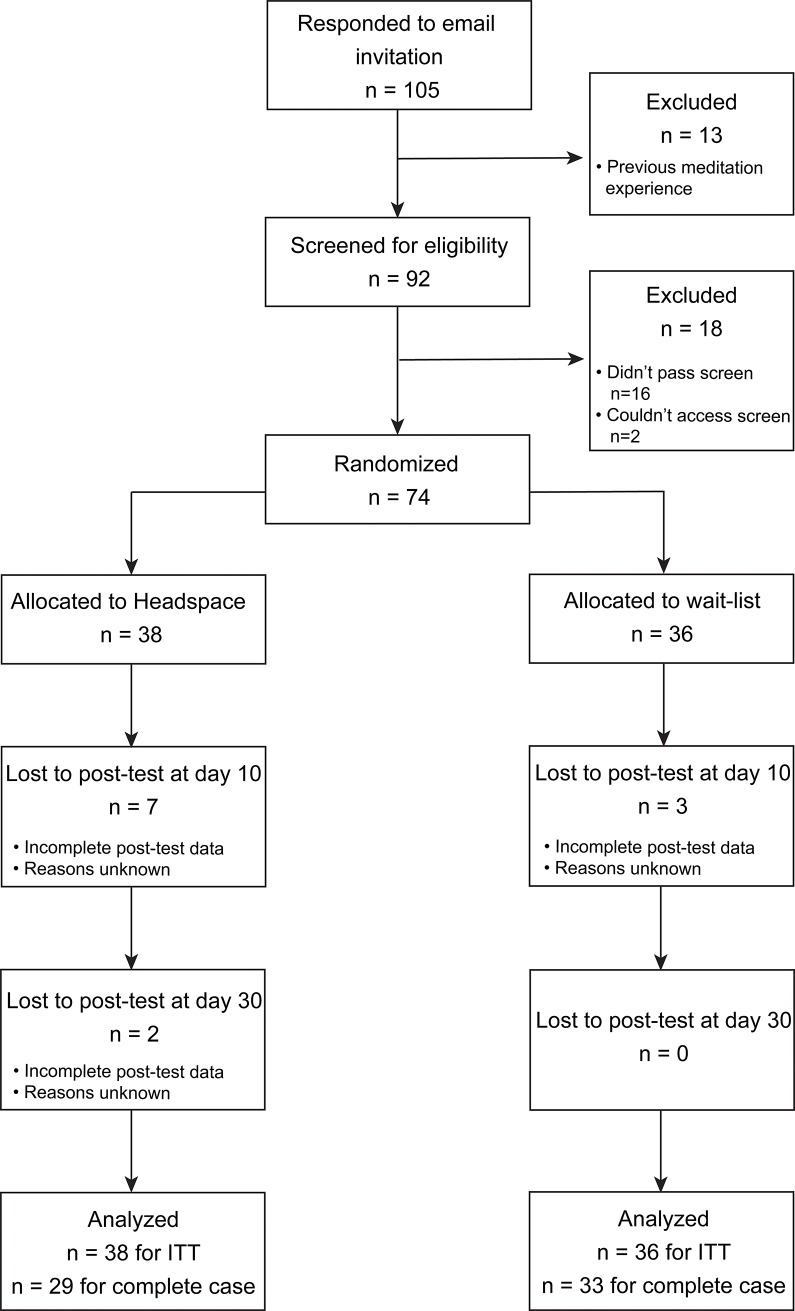
CONSORT diagram of participant flow through the study.

Fig 1 displays the flow of participants through the study. Of the 74 participants randomised, 62 participants completed the intervention and measures at all three time points (n = 29 for the MM group, n = 33 for the WL group). Dropout across all 30 days of the intervention corresponded to 23.7% in the MM group and 8.33% in the WL group, but was not significantly different between groups (*ß*^2^ (1) = 0.28, p = 0.59).

### Baseline equivalence

Age ranged from 25–59 years (M = 39.13, SD = 5.70, 37 females and 25 males; see Table 1 for characteristics of the sample). Note that whilst mean age was similar between groups, randomisation resulted in a larger proportion of females than males in the wait-list group, likely reflecting a limitation of simple randomisation. Baseline scores suggest that participants in both groups had average levels of satisfaction with life (c.f. [37]), average to high levels of stress (c.f. [40]), and moderate levels of resilience (c.f. [48]) prior to engaging with the study. Mean GHQ-28 scores at baseline were 3.72 (SD = 3.03) in the MM group and 3.36 (SD = 3.16) in the WL group. These scores are below the general threshold for possible psychological distress [49], but suggest that at least some of the participants may have been candidates for distress. Scores did not differ between groups at baseline on any measures (two-sample t-tests, all p > 0.05).

**Table 1:** Participant demographics.

	Mindfulness group		Wait-list group	
	Mean	SD	Mean	SD
Age	40.17	4.08	38.21	6.75
Gender	N	%	N	%
Male	16	55.2%	9	27.3%
Female	13	44.8%	24	72.7%
GHQ-28	3.72	3.03	3.36	3.16

Characteristics of the mindfulness meditation and wait-list control groups at baseline.

### Intention-to-treat analysis

Linear mixed effects models revealed significant group x time interactions for satisfaction with life (*ß* = 0.11, SE = 0.035, p = 0.002), perceived stress (*ß* = -0.28, SE = 0.052, p < 0.001), and resilience (*ß* = 0.23, SE = 0.079, p = 0.003). There was significant negative covariance between intercept and slope across participants for perceived stress (-0.77; 95% CI -0.89 to -0.54) and for resilience (-0.61; 95% CI -0.77 to -0.37), and a trend towards negative covariance for satisfaction with life (-0.41; 95% CI -0.71 to 0.006). This suggests that participants with lower initial scores on the SWLS and WRS, or higher initial scores on the PSS, were likely to experience the largest self-reported benefit over time. R^2^ for the winning model was 0.81, 0.77 and 0.92 for SWLS, PSS and WRS respectively.

For each outcome, we used multiple imputation to impute missing values and then calculated a between-group Cohen’s *d* effect size between baseline and day 30. This revealed a Cohen’s *d* of 0.57 (95% CI: 0.10 to 1.04) for satisfaction with life, 1.42 (95% CI: 0.90 to 1.94) for perceived stress, and 0.63 (95% CI: 0.16 to 1.10) for resilience (see Table 2).

**Table 2:** Outcome measures and Cohen’s *d* effect sizes.

Outcome measure	Condition		Baseline mean (SD)	Day 10 mean (SD)	Day 30 mean (SD)	Cohen’s d (95% CI)			
						Within-group		Between-group	
						Baseline to day 10	Baseline to day 30	Baseline to day 10	Baseline to day 30
SWLS	Mindfulness app	Completers only	24.17 (5.39)	26.00 (4.23)	27.69 (4.75)	0.46 (0.14 to 0.78)	0.65 (0.23 to 1.07)	0.32 (-0.19 to 0.83)	0.60 (0.08 to 1.12)
		Imputed values	24.18 (5.73)	26.03 (4.04)	27.70 (4.32)	0.51 (0.25 to 0.77)	0.70 (0.35 to 1.05)	0.30 (-0.16 to 0.76)	0.57 (0.10 to 1.04)
	Wait-list	Completers only	24.55 (5.55)	24.64 (5.44)	24.76 (5.70)	0.03 (-0.17 to 0.23)	0.06 (-0.15 to 0.27)		
		Imputed values	24.56 (5.89)	24.64 (5.60)	24.76 (5.85)	0.03 (-0.15 to 0.21)	0.06 (-0.12 to 0.24)		
PSS	Mindfulness app	Completers only	16.90 (4.86)	14.90 (4.89)	11.41 (5.63)	0.53 (0.21 to 0.85)	0.83 (0.33 to 1.33)	0.16 (-0.35 to 0.67)	1.53 (0.95 to 2.11)
		Imputed values	16.68 (5.25)	14.81 (4.58)	11.40 (4.93)	0.52 (0.26 to 0.78)	0.84 (0.40 to 1.28)	0.14 (-0.32 to 0.60)	1.42 (0.90 to 1.94)
	Wait-list	Completers only	17.73 (5.64)	16.58 (4.74)	20.36 (3.21)	0.27 (-0.02 to 0.56)	-0.47 (-0.03 to -0.91)		
		Imputed values	17.61 (6.01)	16.51 (4.76)	20.35 (3.09)	0.26 (0.00 to 0.52)	-0.47 (-0.06 to -0.88)		
WRS	Mindfulness app	Completers only	73.69 (11.64)	76.97 (10.53)	81.86 (10.14)	0.64 (0.40 to 0.88)	0.79 (0.39 to 1.19)	0.17 (-0.34 to 0.68)	0.61 (0.09 to 1.13)
		Imputed values	74.68 (11.41)	77.92 (10.05)	82.44 (9.15)	0.71 (0.50 to 0.92)	0.83 (0.49 to 1.17)	0.17 (-0.29 to 0.63)	0.63 (0.16 to 1.10)
	Wait-list	Completers only	75.00 (11.12)	76.39 (11.92)	76.24 (9.10)	0.20 (-0.01 to 0.41)	0.14 (-0.17 to 0.45)		
		Imputed values	75.86 (11.14)	77.15 (11.77)	76.66 (8.85)	0.20 (0.00 to 0.39)	0.09 (-0.20 to 0.38)		

Baseline, day 10, and day 30 scores (with corresponding Cohen’s *d* effect sizes) for the SWLS, PSS and WRS in the mindfulness meditation group and wait-list control group. For the complete case: n = 29 for the mindfulness group and n = 33 for the wait-list control; when including imputed values: n = 38 for the mindfulness group and n = 36 for the wait-list control.

### Complete case analysis

#### Satisfaction with life

A 2 x 3 ANOVA revealed a main effect of time (*F*(2,120) = 5.74, p = 0.004) and a group by time interaction for the SWLS (*F*(2,120) = 5.057, p = 0.008). Post-hoc paired t-tests showed that satisfaction with life increased in the MM group from baseline to day 10 (*t*(28) = 2.39, p = 0.024), and increased further from day 10 to day 30, though the latter just failed to reach significance (*t*(28) = 1.84, p = 0.076) (see Table 2 and S1 Fig). By contrast, satisfaction with life did not increase in the WL group across any of the assessment points (all p > 0.05). A between-group effect size analysis revealed a Cohen’s *d* of 0.60 (95% CI: 0.08 to 1.12) between baseline and day 30 (see Table 2). When examining individual participants in the MM group, 20 (69%) reported subjective increases in satisfaction with life, 6 (21%) reported no change, and 3 (10%) reported decreases in satisfaction with life following day 30 of the intervention (see S2 Fig).

#### Perceived stress

A similar pattern emerged for perceived stress, with a 2 x 3 ANOVA revealing a significant group by time interaction (*F*(2,120) = 21.98, p < 0.001). A main effect of group (*F*(1,120) = 15.56, p < 0.001), and an almost significant main effect of time (*F*(2,120) = 2.88, p = 0.06) also emerged. Post-hoc paired t-tests showed that perceived stress decreased in the MM group between baseline and day 10 (*t*(28) = 2.84, p = 0.008), and decreased further between day 10 and day 30 (*t*(28) = 3.16, p = 0.004) (see Table 2 and S1 Fig). There were no changes in stress between baseline and day 10 in the WL group (p > 0.05). However, in contrast to the MM group, stress unexpectedly increased between days 10 and 30 (*t*(32) = 4.49, p < 0.001). A between-group effect size analysis revealed a Cohen’s *d* of 1.53 (95% CI: 0.95 to 2.11) between baseline and day 30 (see Table 2). When examining individual participants in the MM group, 23 (79%) reported subjective decreases in perceived stress, while 6 (21%) reported a subjective increase in stress, following day 30 of the intervention (see S2 Fig).

#### Resilience

A 2 x 3 ANOVA revealed a main effect of time (*F*(1,120) = 9.21, p < 0.001) and a group by time interaction (*F*(1,120) = 5.86, p = 0.004) when analysing WRS scores. Post-hoc paired t-tests showed that resilience scores in the MM group increased between baseline and day 10 (*t*(28) = 3.37, p = 0.002), and increased further between day 10 and day 30 (*t*(28) = 2.96, p = 0.006) (see Table 2 and S1 Fig). Resilience scores did not differ between any assessment points in the WL group (all p > 0.05). A between-group effect size analysis revealed a Cohen’s *d* of 0.61 (95% CI: 0.09 to 1.13) between baseline and day 30 (see Table 2). When examining individual participants in the MM group, 21 (72%) reported subjective increases in resilience, 2 (7%) reported no change in resilience, while 6 (21%) reported subjective decreases in resilience following day 30 of the intervention (see S2 Fig). Note that 4 (14%) participants reported a subjective decrease in well-being across two of the three outcomes, whilst no participant reported a decrease in well-being across all three outcomes.

### Engagement and task enjoyment

Participants in the MM group reported engaging with the app an average of 6.21 times (SD = 2.65) between baseline and day 10 (min = 1, max = 10), with 22 out of 29 participants completing 5 or more out of the 10 sessions. Participants reported engaging with the MM app 11.66 times (SD = 6.16) between day 11 and 30 (min = 1, max = 20), with 17 out of 29 participants completing 10 or more out of the 20 sessions. Six participants completed 25 or more out of a maximum of 30 sessions. All participants reported selecting a session duration of 10 minutes throughout the intervention, except for one participant that reported increasing the duration to 15 minutes from session 11 (level 2 of the program) onwards. Over 75% of participants rated task (intervention) enjoyment as 5 or more (1 = not at all enjoyable, 7 = extremely enjoyable) at day 10, while 69% of participants chose a rating of 5 or more at day 30 (see Table 3). Subjective perception of task difficulty was varied, with most participants rating the intervention as neither very difficult nor very easy (see Table 3).

**Table 3:** Self-reported task enjoyment and difficulty in the mindfulness group.

Self-reported rating	Enjoyment [n (%)]		Difficulty [n (%)]	
	Base–Day 10	Day 10–30	Base–Day 10	Day 10–30
1	0 (0)	1 (3.45)	1 (3.45)	1 (3.45)
2	2 (3.23)	2 (6.90)	3 (10.34)	3 (10.34)
3	1 (1.61)	1 (3.45)	5 (17.24)	8 (27.59)
4	4 (6.45)	5 (17.24)	7 (24.14)	7 (24.13)
5	12 (19.35)	8 (27.59)	6 (20.69)	1 (3.45)
6	7 (11.29)	8 (27.59)	3 (10.34)	5 (17.24)
7	3 (4.84)	4 (13.79)	4 (13.79)	4 (13.79)

Self-reported ratings of intervention enjoyment and difficulty in the mindfulness meditation group. Enjoyment and difficulty were rated between baseline and day 10, and between day 10 and day 30. 1 = ‘Not at all enjoyable’ (for enjoyment), or ‘Very difficult’ (for difficulty); 7 = ‘Extremely enjoyable’ (for enjoyment), or ‘Very easy’ (for difficulty).

Participants that rated the intervention as being relatively easy between days 10 and 30 were likely to have larger increases in satisfaction with life (*ß* = 1.264, p = 0.006), larger decreases in stress (*ß* = 1.471, p = 0.015), and larger increases in resilience (*ß* = 2.570, p = 0.003) over the same time period. Further, participants with higher GHQ-28 scores at baseline (higher levels of baseline distress) were likely to have larger increases in satisfaction with life (*ß* = 0.508, p = 0.041) and larger increases in resilience (*ß* = 1.231, p = 0.009) between days 10 and 30 of the MM intervention. Lastly, older participants were likely to have a larger increase in satisfaction with life between baseline and day 10 (*ß* = 0.323, p = 0.049), whilst females were likely to experience larger decreases in stress between day 10 and day 30 than males (*ß* = 4.794, p = 0.019).

## Discussion

While the evidence-base supporting traditional in-person MM training is now substantial [8,50], there is increasing popularity and empirical support for online MM interventions in both clinical and non-clinical settings [16]. However, few studies have investigated the efficacy of mindfulness-based smartphone apps, which are rapidly increasing in popularity. The current pilot study investigated whether using a commercial MM app increases aspect of psychosocial well-being in healthy adults over a period of 10 or 30 days. Relative to a WL control group, 10 days of the MM app positively impacted self-reported satisfaction with life, perceived stress, and resilience, with the magnitude of impact increasing further after 30 days. Although limited by a small sample size and an inactive control group, the present study is one of the first pilot RCTs to investigate the impact of a MM app on well-being over a 30-day period, and suggests that short-term use of a MM app has the potential to improve self-reported psychological health in self-selected participants from the general population. These findings should be followed up with more substantive, larger-scale RCTs.

Whist the findings reported here are largely consistent with a recent meta-analysis that online MM interventions increase aspects of psychosocial well-being [16], only nine studies included in the meta-analysis measured well-being, and even fewer studies have specifically measured well-being following an app-based MM intervention. Although online and app-based interventions share features that make them distinct from in-person training, commercial MM apps may substantially diverge from online interventions (which are frequently modelled after MBSR and other in-person programs), and therefore should be evaluated separately. One recent app-based MM study was conducted by Howells et al. (2014), and examined the efficacy of the same MM app as the present study over a short-term 10-day period. The study showed that 10 days of app-based MM increased self-reported positive affect and reduced depressive symptoms in a cohort of ‘happiness seekers’, but not did significantly impact satisfaction with life [21]. This contrasts with the present finding that 10 days of app-based MM was sufficient to significantly increase self-reported satisfaction with life. This discrepancy may have been driven by differences in study sample characteristics (88% of participants in Howells et al. were female), differences in baseline levels of psychological well-being (participants reported lower levels of satisfaction with life, and higher than average levels of negative affect at baseline in Howells et al.), differences in control groups (Howells et al. utilised an active control), or differences in app engagement. However, whilst the within-group effect size for satisfaction with life was significant at days 10 and 30 in the MM group, the between-group effect size was only meaningful at day 30, suggesting that the difference between study groups was only robust at the end of the intervention. Other recent studies have reported that engaging with a MM app for 8 weeks can reduce general psychiatric symptoms, including symptoms of depression [51], and increase quality of life in healthy adults [20].

The present study found medium effect sizes for self-reported satisfaction with life and resilience, and a large effect size for perceived stress following 30 days of MM, albeit with wide confidence intervals. The fact that perceived stress showed the largest effect is consistent with a recent meta-analysis of online MM interventions [16]. Although the between-group effect size for stress (*d* = 1.42) was likely inflated by an inadvertent increase in stress in the WL group, the within-group effect size (*d* = 0.84) for the MM group was very close to that reported by a recent meta-analysis of MBSR interventions for healthy adults (*g* = 0.83; [8]). Whilst this is reassuring, it is somewhat unexpected given substantial differences between MBSR and the MM app used in the present study (MBSR is delivered in-person, is group-based, is conducted over 8 weeks, and involves a much higher total volume of home practice). This may suggest that mindfulness training causes a fairly rapid reduction in stress, beyond which further reductions occur at an exponentially slower rate. However, we caution readers not to overinterpret these results, given the small sample size, the lack of an active control group, and the inclusion of self-selected participants in the present study. Although few previous MM studies have utilised the SWLS and the WRS, one recent study reported a medium effect size for satisfaction with life following a web-based MM intervention [52], consistent with the effect reported in the present study.

An important but understudied question is how much mindfulness practice is needed in order for benefits to emerge [53]. Whilst some studies suggest that just a single meditation session is sufficient to increase state mindfulness [54] and positive affect [55], others suggest that improvements in stress require a minimum of 4 weeks of training [56]. In the present study, participants experienced consecutive increases in self-reported well-being following 10 and 30 days, though the rate of benefit was greatest between baseline and day 10, during which participants completed an average of just 6.21 meditations. This suggests that in healthy participants, MM training has a rapid and measurable impact on subjective well-being, with the rate of benefit modestly subsiding by day 30 of the present study. This is consistent with other recent studies in healthy participants showing improvements in stress, affect and irritability [19], and symptoms of anxiety/depression [57], following online MM interventions as short as just two weeks. However, we caution readers that the between-group effect size for all three outcomes was only significant following day 30 of the intervention, and future studies with larger sample sizes are needed to order to determine the rate of benefit following app-based MM interventions with greater precision. If MM apps *are* able to impart fairly immediate benefits, this would have implications for reducing the societal burden of stress-related illness, and increasing the health of individuals, organisations and national economies, especially given the low cost and scalability of mobile health interventions relative to in-person programs.

Previously, Howells et al. (2014) reported that the largest increase in positive affect following 10 days of app-based MM occurred in participants that reported the highest levels of intervention satisfaction. In the present study, a similar finding emerged wherein participants that rated the intervention as easy to engage with were likely to experience the largest benefits. This association could partly be driven by higher engagement rates in participants that rated the intervention as enjoyable (as engagement and task difficulty were highly correlated). However, this finding also raises the question of whether self-guided MM training is more suited to certain individuals over others. A recent study found that participants with a lower initial severity of depressive symptomatology were likely to benefit from smartphone-based MM training more than those with a higher initial severity of depression [58]. In the present study, participants with higher levels of distress at baseline were likely to experience larger increases in satisfaction with life and resilience. Together, these results raise a potential opportunity for the development of app-based interventions that can be adapted according to the needs of specific populations or individuals. For example, factors such as session length, the specific set of techniques used, and the volume of instruction and support, could all be adjustable in an effort to boost overall effectiveness.

Whilst perceived stress decreased in the MM group across all three assessment points, stress unexpectedly increased in the WL group at the third assessment point (day 30). A likely explanation for this is that the third assessment point fell approximately 24 hours after the result of the Brexit European Union Referendum was announced in the UK. Since all participants took part during the same 30-day period, and the majority of participants were likely based in London (a predominantly anti-Brexit constituency), it’s plausible that this event could have triggered an increase in stress. If this is indeed the case, then the decrease in stress observed in the MM group suggests that MM training not only has the potential to reduce stress in the context of day-to-day life, but also to protect against stress induced by adverse events. Despite this positive result, 4 out of 29 participants in the MM group reported a decrease in subjective well-being across 2 of the 3 outcomes. This may have been driven by measurement noise, factors outside of experimental control (such as the Brexit vote), or due to the MM app being ineffective or harmful. Whilst it’s not possible to differentiate between these causes, there’s currently little evidence to suggest that mindfulness training of up to 20 minutes per day can be harmful for healthy individuals, and no participants reported a decrease in well-being across all 3 outcomes.

An exploratory analysis revealed that females were likely to experience larger decreases in stress across the MM intervention than males. Females were marginally less stressed and more resilient than males at baseline, and on average completed 19 meditations compared to 17 for males, though none of these differences were statistically significant. A recent study found that women benefited more from a school-based MM program more than men, with the authors positing that this may be due to gender-based differences in emotion regulation techniques [59]. Previous evidence suggests that women tend to internalize negative emotion through rumination, whilst men externalise negative emotion through external distraction [60,61]. One possibility, proposed by Rojiani and colleagues, is that MM may address women’s tendency to internalise stress [59], given that MM has previously been shown to reduce rumination [62,63]. Our analysis also showed that older participants were likely to experience a larger subjective increase in satisfaction with life between baseline and day 10 of the intervention. However, older participants had significantly lower satisfaction with life scores at baseline, which may be responsible for this finding. Given the exploratory nature of these analyses, the small sample size, and narrow age range in the present study, we caution readers not to overinterpret these results. Further research is needed to understand the roles that gender and age play in the efficacy of MM interventions.

### Limitations

The current study has a number of limitations. First, although we attempted to recruit a sample size in keeping with guidelines for pilot RCTs [27], our outcome measures were assessed online, and relied on self-reported questionnaires, which are noisy and open to interpretation [64]. Thus, future studies may wish to compensate for this potential bias by recruiting larger samples.

Second, in an effort to reduce the length of our assessments (and thus reduce attrition), whilst capturing as many outcomes that relate to well-being as possible, mindfulness was not included as an outcome measure. It therefore remains unknown whether the change in outcome scores were mediated by an increase in trait mindfulness or by another mechanism. However, three previous studies that utilised the same MM app as the present study have reported increases in mindfulness [31,32], one of which utilised the exact same intervention as the current study [33]. Thus, it seems plausible that mindfulness may have increased in the present study. A number of previous studies have directly linked increases in mindfulness to improvements in psychosocial well-being [65,66].

Third, the present study did not investigate the efficacy of the MM app beyond 30 days, nor whether any of the findings were maintained (regardless of app usage) beyond this period. It should be noted however, that other studies investigating the efficacy of app-based mindfulness training reported benefits that were maintained for at least 3 [20] to 6 [58] months.

Fourth, whilst attrition in the mindfulness group was relatively favourable compared to previously reported averages in mindfulness research (23.7% compared to 29%; c.f. [67]), high rates of attrition can have important implications. Although the present study utilised the best methods available to handle missing data (such as mixed effect modelling and multiple imputation; [68]), such methods may exaggerate or underestimate treatment effects when attrition is greater than 20% [69]. In addition, since participants were not queried on their reasons for discontinuing usage of the app, the researchers cannot exclude the possibility that some participants in the MM group may have experienced adverse effects that went unreported. Such adverse effects have previously been raised as a concern in meditation research [70], though to the best of our knowledge only one prospective study has reported negative effects following an intense meditation retreat consisting of over 10 hours of practise per day [71]. Thus, as previously stated, there’s little evidence to suggest that MM techniques practised for up to 20 minutes per day can induce harm in healthy individuals. Nonetheless, understanding the factors responsible for attrition could help researchers improve the design and efficacy of future interventions, as well as increase our understanding of adverse effects following MM, which is as yet substantially understudied.

Fifth, although there were no differences between groups with regards to stress, resilience and satisfaction with life at baseline, randomisation resulted in a larger proportion of females than males in the wait-list group. This reflects a limitation of utilizing simple randomisation, and future studies should attempt to avoid such confounds by using other methods of randomisation (such as stratified), in combination with larger sample sizes.

Sixth, whilst the sampling methods used to recruit study volunteers was quick and convenient, it has a number of disadvantages. Participants were self-selected and likely came from similar social or cultural backgrounds that may not necessarily represent the general population. Studies that don’t use random sampling methods have an increased chance of sampling bias and the potential for certain findings to be understated or overstated [72]. Although it is impossible to predict whether and how the sampling methods used in the present study impacted the findings, future research should attempt to replicate the results of this study in other cohorts and using random sampling methods.

Seventh, app engagement was not objectively measured, but was derived from self-reported data, which is inherently unreliable. Having an accurate understanding of the effective dose, or total volume of meditation sessions completed, is important for understanding both the effectiveness and appeal of the intervention, particularly in cases where app usage can vary considerably between participants.

Eighth, the research team was not formally blinded to group allocation. Although we are confident that this has not influenced the analysis or findings, future studies should rule out this possibility by formally blinding the statistical analysis to group allocation.

Ninth, a wait-list control condition was used as opposed to an active control. A failure to utilize rigorous control or comparison conditions has previously been discussed as a major limitation in meditation research [73]. Future studies evaluating app-based mindfulness interventions should attempt to replicate these and other results using active control groups.

Tenth, although most study participants were located in the UK, participant race and ethnicity were not directly measured. This is important because race and ethnicity may have a moderating impact on intervention engagement and/or efficacy. Studies that predominantly recruit individuals of the same race or ethnicity may therefore be less generalizable.

Lastly, the present study targeted employees from the general population in an attempt to investigate whether Howells and colleagues’ findings in happiness seekers would extend to the general public. However, the study was framed as an investigation into mindfulness and well-being, and this may have biased the study cohort. Though satisfaction with life scores were higher at baseline in the present study than in Howells’, GHQ-28 scores at baseline suggest that at least some participants may have been experiencing higher than average levels of distress. Further, participants with higher GHQ-28 scores at baseline were likely to experience larger increases in satisfaction with life and resilience post-intervention. Thus, caution should be exercised when attempting to generalise the findings reported here to other cohorts. With MM experiencing a dramatic increase in media coverage, it is becoming increasingly difficult to recruit naïve participants. However, future studies should aim to systematically investigate the impact of app-based MM training in more diverse, less biased study populations.

## Conclusions

Research investigating the efficacy of self-guided MM training via smartphone apps is scarce, despite a rapid increase in the popularity of such apps. The present pilot study suggests that 10 days of using a commercial MM app is sufficient to induce significant positive impact on self-reported stress, resilience, and satisfaction with life, with the magnitude of benefit increasing further following 30 days. These results support recent evidence that mobile apps are a cost-effective, highly accessible, and suitable medium for the delivery of well-being interventions. More substantive research is needed to replicate and extend these findings in longer interventions, larger cohorts, and more diverse populations.

## Supporting information

S1 Checklist CONSORT checklist. CONSORT 2010 checklist of information to include when reporting a randomised controlled trial.
(DOC)

S1 Fig Outcome scores. Outcome scores for all three measures, at all timepoints, in the mindfulness meditation group (n = 29) and wait-list control (n = 33) group. Error bars correspond to SEM.
(TIF)

S2 Fig Change in outcome scores for individual participants. Change in score for each individual participant for all three measures between baseline and day 30 in the mindfulness group. Each bar corresponds to one participant (n = 29). Participants who experienced a score change in the beneficial direction are represented in blue, whilst those who experienced a score change in the harmful direction are represented in amber.
(TIF)

S1 Dataset Raw data. Anonymised data for the mindfulness meditation and wait-list control group including sociodemographic data and outcome data across all three timepoints.
(XLSX)

S1 Protocol Study protocol with invitation and briefing emails. Original study protocol as approved by the London Metropolitan University ethics committee, in addition to the study invitation email and briefing emails for both the mindfulness meditation and wait-list control groups.
(DOCX)
