# Supplementary figures and images for: The efficacy of a brief app-based mindfulness intervention on psychosocial outcomes in healthy adults: A pilot randomised controlled trial
Source: PLoS One. 2018 Dec 31;13(12):e0209482. doi: 10.1371/journal.pone.0209482 (PMC6312207; doi:10.1371/journal.pone.0209482)

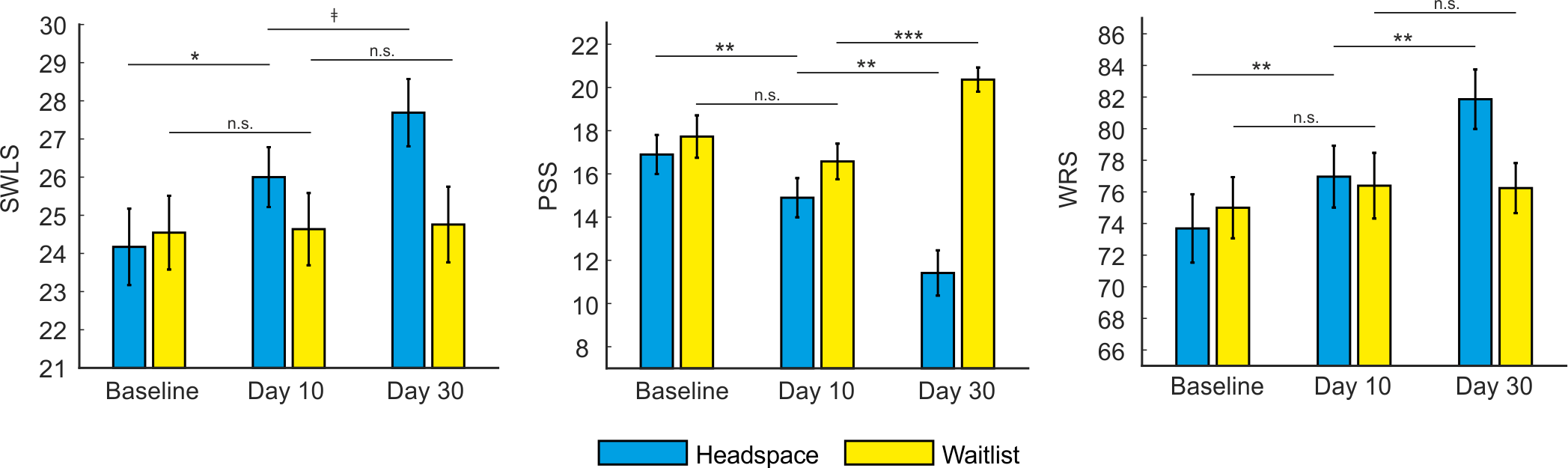

Supplement: S1 Fig — Outcome scores for all three measures, at all timepoints, in the mindfulness meditation group (n = 29) and wait-list control (n = 33) group. Error bars correspond to SEM. (TIF) [file pone.0209482.s002.tif]

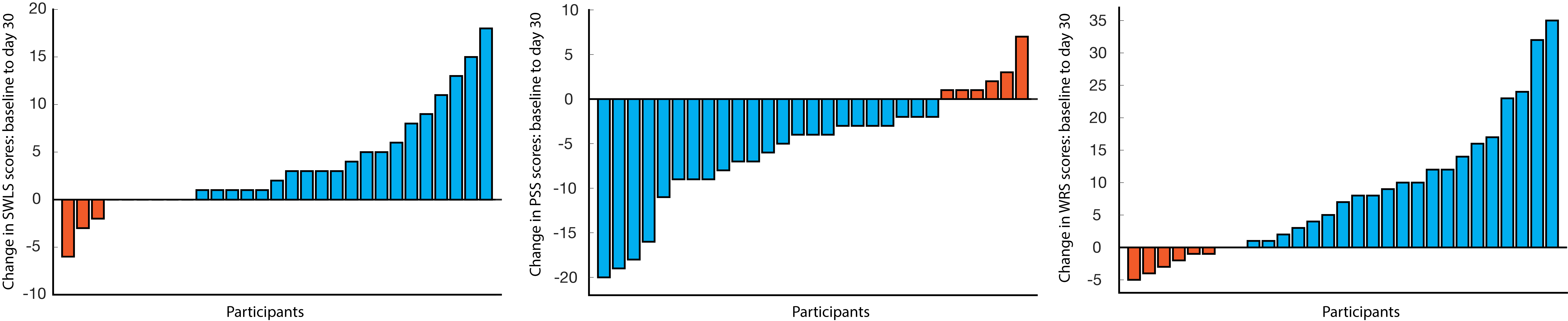

Supplement: S2 Fig — Change in score for each individual participant for all three measures between baseline and day 30 in the mindfulness group. Each bar corresponds to one participant (n = 29). Participants who experienced a score change in the beneficial direction are represented in blue, whilst those who experienced a score change in the harmful direction are represented in amber. (TIF) [file pone.0209482.s003.tif]
