# Supplementary material for: The efficacy of a brief app-based mindfulness intervention on psychosocial outcomes in healthy adults: A pilot randomised controlled trial
Source: PLoS One. 2018 Dec 31;13(12):e0209482. doi: 10.1371/journal.pone.0209482 (PMC6312207; doi:10.1371/journal.pone.0209482)
Supplement: S1 Protocol — Original study protocol as approved by the London Metropolitan University ethics committee, in addition to the study invitation email and briefing emails for both the mindfulness meditation and wait-list control groups. (DOCX) [file pone.0209482.s005.docx]

**Background/rationale**

Technology is advancing and becoming smarter. The smartphone plays a key role in this advancement, with estimates suggesting that 1.82 billion smartphones were globally in use at the end of 2013 (Khalaf, 2013). Aside from communication, smartphones have numerous other applications or functions that can be accessed via ‘apps’. This variety has promoted a dependency on smartphone use, with estimates suggesting that users check their devices every 6.5 minutes on average (Khalaf, 2013). This increase in use highlights the potential for smartphones to be used as a means to disseminate psychological interventions to the general public, in a cost-effective and widely accessible manner.

Positive psychology models suggest that happier people are more likely to acquire favourable life circumstances, which in turn is likely to engender greater happiness in the long-term (Lyubomirsky et al., 2005). Specifically, investing in ones’ own well-being has been linked to marked benefits across a range of characteristics and resources most valued by society, such as longer life expectancy, higher earnings, and better relationships (Lyubomirksy et al., 2005). For smartphone users looking to increase their happiness, there are now thousands of applications, or ‘apps’, available to them. Smartphone apps may be more accessible, flexible, interactive, and private than other forms of self-help (Howells, Ivtzan, Eiroa-Orosa, 2014). However, despite these potential advantages, few studies have empirically tested whether smartphone apps are an effective means of increasing well-being in the general public (Howells, Ivtzan, Eiroa-Orosa, 2014).

One particular psychological tool that has shown promise as a means of increasing well-being is mindfulness. Mindfulness involves focusing on the present moment, in a non-judgemental, non-reactive way (Kabat-Zinn,1994). The practice has historically been associated with Buddhist teachings dating back several thousand years, and was introduced to the West in the 1970’s (Chiesa & Malinowski, 2011). Since then, a wealth of scientific research has outlined a variety of positive impacts of mindfulness meditation on health and well-being, including reductions in cognitive vulnerability and emotional distress (Shapiro and Carlson, 2009, Baer 2003). Further, multiple studies have linked the practice of mindfulness with increased quality of life and reduced stress (Reibel, Greeson, Brainard, & Rosenzweig, 2001; Shapiro, Schwartz, & Bonner, 1998; Williams, Kolar, Reger, & Pearson, 2001). Mindfulness meditation is also the cornerstone of the well-known Mindfulness Based Stress Reduction Programme (MBSR), originally designed to treat a range of conditions in both clinical and non-clinical populations. MBSR typically takes the form of an 8-week, in-person, group-based programme, coupled with approximately 45 minutes of home practice per day.

Meta-analyses have linked mindfulness training to changes in brain regions associated with attention, memory, self and emotional regulation (Fox et al., 2014). Organisations such a Google and the US army use mindfulness training to improve work place functioning (Jha et al., 2015). The rationale for such interest is based on evidence that mindfulness impacts positively on human functioning (Brown, Ryan & Creswell, 2007). Various mechanistic models have been presented in an attempt to outline how mindlessness-based interventions convey such benefits. Vago and David’s (2012) self-awareness, self-regulation and self-transcendence model (S-ART) provides a theoretical framework based on a neurobiological model describing how mindfulness reduces biases to self-processing and promotes psychological health. Shapiro’s model (2006) outlines the fundamental change in an individual’s relationship to experience (perceiving) following mindfulness training, which leads to changes in self-regulation, and increased cognitive and behaviour flexibility. From a more evolutionary perspective, Williams (2010) suggests mindfulness brings about changes in the way emotions are processed, guiding individuals on how to learn to discriminate between different emotions.

In a recent study, Howells, Ivtzan and Eirosa (2014) examined the impact of a smartphone-based intervention on measures relating to well-being. Their randomised controlled trial examined the impact of 10 days of a Headspace mindfulness intervention using a cohort of ‘happiness seeking’ participants, and measured satisfaction with life, flourishing, positive and negative affect, and symptoms of depression. Relative to a control group, they reported statistically significant increases in positive affect and reductions in depressive symptoms following 10 days of Headspace use, but did not find statistically significance benefits with regards to life satisfaction, flourishing or negative affect scores.

The present study aims to both replicate and extend Howells and colleagues study by investigating the impact of Headspace over a longer (30-day) period, while measuring life satisfaction, stress, and resilience, in a cohort of healthy participants from the general population. The study aims to test whether a smartphone-based mindfulness intervention, delivered via the Headspace app, can lead to significant improvements in psychosocial outcome measures over a longer intervention period.

**Aims/objectives**

This study aims to identify:

- If self-reported measures of stress, life satisfaction, and resilience, improve after 10 and 30 days of a mindfulness-based app (Headspace) intervention, relative to a wait-list control group.

- If there is a linear or non-linear change in scores between baseline, day 10, and day 30 in the experimental condition.

- If those who experience the largest reductions in stress also experience the largest increases in resilience.

**Hypotheses**

H1: The control group will have significantly worse scores on all measures at day 10 and day 30 day compared to the experimental (Headspace) group.

H2: There will be a greater positive impact on study outcomes following 30 days of Headspace use relative to 10 days.

H3: Those who experience larger reductions in stress across 30 days of the Headspace intervention will also experience larger increases in resilience over the same time period.

**Research Methodology**

This study will use a repeated-measures, between-group, randomised controlled design.

Participants will only be selected if they indicate good general health, no prior experience with mindfulness practice, and no recent adverse life events, as assessed via an initial screening tool. Those who meet inclusion criteria and respond within the recommended time period of two weeks will be directed to a webpage, via the Survey Monkey platform, and a detailed participant information sheet. Participants will be informed that they will be randomly assigned either to an experimental group or to a ‘wait-list’ control group, and will be asked to provide informed consent before completing a brief demographics questionnaire. Participants will then be randomly allocated to the Headspace intervention or to a control group. The control group will be informed that they are a ‘wait-list’ group, and will be required to complete questionnaire measures at baseline, and 10 and 30 days later, before being offered access to the Headspace intervention for a further 30 days (should they wish to use it). They will be given detailed instructions of how to download and use the Headspace intervention via their smartphone at the end of the wait-list period. The treatment group will be given instructions to complete questionnaire measures at baseline and then again at 10 and 30 days of the Headspace intervention. Following baseline measures, participants in the Headspace group will be given an access code and details of how to download the Headspace programme to their smartphone. Participants will be gently encouraged to use the app every day during the 30-day study period, and to retain a diary to record hours/days of activity/usage. Between days 9 and 10 of the Headspace intervention, participants in both groups will receive an email encouraging them to complete all outcome measures again via Survey Monkey. Another email reminder will be sent out within 24 hours of completing the full 30-day intervention. If no action is taken further emails will be sent within 5 days of completion. Participants in both groups will be reminded they have the right to withdraw at any point during the study.

**Materials**

*Screening Tool*

The initial screening tool has been partly designed by the researchers, though some items have been drawn from existing screening tools, as well as recommendations from Kuyken, Crane and Williams (2012) within their paper on MBCT Implementation. Recent pilot work conducted by City University (Bogosian et al., 2016) investigating an online-based mindfulness intervention for multiple-sclerosis patients recommends the use of the General Health Questionnaire as a screening tool in mindfulness research. Additional screening items will include questions regarding the presence of any serious psychological disorders (such as psychosis and substance abuse, post-traumatic stress disorder, depression), prior or current attendance of a psychological therapy programme, prior formal training or engagement with mindfulness programmes and meditation, the presence of any recent or foreseeable difficult life events (such as a divorce, or moving house), and the presence of any physical illness that is likely to cause acute pain or discomfort. Many of these may impede engagement with the intervention and therefore impair the emergence of any potential benefits associated with it. Participants will also be asked to provide basic sociodemographic information (age and gender).

The General Health Questionnaire 28 (Goldberg, 1978) is a 28-item measure specifically designed as a screening tool and to assess four factors of distress over the previous two weeks – depression, anxiety, social impairment and hypochondriasis. Test-retest reliability has been reported to be high (0.78 to 0.9) (Robinson & Price, 1982) and interrater and intrarater reliability have both been shown to be excellent (Cronbach's α 0.9–0.95) (Failde and Ramos 2000). Responses 1 and 2 (not at all/no more than usual) are assigned a score of zero, whereas a score of 1 is assigned to “rather” responses - more than usual/much more than usual – as per original authour recommendations. Total scores exceeding 4 out of a possible 28 will be excluded from the research as this suggests probable distress.

*Outcome Measures:*

The Satisfaction with Life Scale (1985) is a narrowly focused scale to access global satisfaction. It is shown to have strong psychometric properties including internal consistency and high temporal reliability (Diener, Emmons, Larsen, & Griffin, 1985). It consists of five brief statements and respondents are instructed to give answers using a seven-point Likert Scale.

The Perceived Stress Scale (1983) is a 10-item scale designed to measure the degree to which situations in an individual’s life might be interpreted as stressful. Respondents answer questions about their feelings and thoughts using a 5-point Likert scale (0=never to 5=very often). It has high validity and test-retest reliability (Cohen, Kamarck, Mermelstein, 1983).

The Wagnild Resilience Scale (1993) is a 14-item scale designed to measure five characteristics of resilience. Respondents answer using a 7-point Likert Scale. The scale has good internal consistency, internal reliability, and construct validity (Wagnild, 1993).

An evaluation scale based on Lyubomirsky & Layous, 2013. This measure seeks to assess the number of days of completed activity along with the extent to which participants enjoy the activity and to the extent to which any difficulties are encountered.

**Recruitment of Participants**

Opportunistic and snowball sampling will be applied via an email asking for volunteers from the general population to take part in a study on mindfulness and well-being (see Appendix for email copy). Those that respond within a two-week time period will be guided to a webpage on Survey Monkey, which will introduce the research and screening procedure. If the participant passes the screening tool, they will be randomly assigned to an experimental group or a control group. The target sample size is 60 participants in total. For those who do not meet inclusion/exclusion criteria, they will be immediately debriefed and further guidance provided.

**Statistical Analysis**

Analysis will be carried out using repeated measures analysis of variance (ANOVA) with group (mindfulness vs control) and time as factors (baseline, day 10, day 30). Pearson’s correlational analysis will also be conducted to explore whether potential gains in well-being experienced by the experimental group are related to participants’ subjective ratings of task enjoyment and task difficulty. Post-hoc t-tests will also be conducted to assess mean score differences between the three time points in each group.

**References**

Baer, R. A. (2003). Mindfulness Training as a Clinical Intervention: A Conceptual and Empirical Review. Clinical Psychology Science and Practice, 10(2), 125-143.

Bogosian, A., Chadwick, P., Windgassen, S., Norton, S., McCrone, P., Mosweu, I., Silber, E. & Moss-Morris, R. Distress improves after mindfulness training for progressive MS: a pilot

randomised trial. Multiple Sclerosis Journal, 21(9), pp. 1184-1194. doi: 10.1177/1352458515576261

Brown, K. W., Ryan, R. M., & Creswell, J. D. (2007). Mindfulness: Theoretical foundations and evidence for its salutary effects. Psychological inquiry, 18(4), 211-237.

Chiesa, A., & Malinowski, P. (2011). Mindfulness‐based approaches: are they all the same?. Journal of clinical psychology, 67(4), 404-424.

Cohen, S., Kamarck, T., Mermelstein, R. (1983). A global measure of perceived stress. Journal of Health and Social Behavior, 24, 385-396.

Diener, E., Emmons, R. A., Larsen, R. J., & Griffin, S. (1985). The Satisfaction with Life

Scale. Journal of Personality Assessment, 49, 71-75.

Failde, I., Ramos, I., & Fernandez-Palacín, F. (2000). Comparison between the GHQ-28 and SF-36 (MH 1-5) for the assessment of the mental health in patients with ischaemic heart disease. Eur J Epidemiol, 16(4): 311-6.

Fox, K. C., Nijeboer, S., Dixon, M. L., Floman, J. L., Ellamil, M., Rumak, S. P., ... & Christoff, K. (2014). Is meditation associated with altered brain structure? A systematic review and meta-analysis of morphometric neuroimaging in meditation practitioners. Neuroscience & Biobehavioral Reviews, 43, 48-73.

Goldberg, D (1978) Manual of the General Health Questionnaire. NFER-Nelson, Windsor

Howells, A., Ivtzan, I., & Eiroa-Orosa, F. J. (2014). Putting the ‘app’in Happiness: A Randomised Controlled Trial of a Smartphone-Based Mindfulness Intervention to Enhance Wellbeing. Journal of Happiness Studies, 1-23.

Jha, A. P., Morrison, A. B., Parker, S. C., & Stanley, E. A. (2016). Practice Is Protective: Mindfulness Training Promotes Cognitive Resilience in High-Stress Cohorts. Mindfulness, 1-13.

Khalaf, S. (2013). Flurry Five-Year Report: It’s an App World. The Web Just Lives in It. Retrieved on August 21, 2013, from: http://blog.flurry.com/bid/95723/Flurry-Five-Year-Report-It-s-an-App-World-The-Web-Just-Lives-in-It

Kabat-Zinn, J. (1994). Wherever you go, there you are: Mindfulness meditation in everyday life. New York:Hyperion.

Kuyken, W., Crane, R., Williams, M (2012) Mindfulness -Based Cognitive Therapy (MBCT) Implementation Resources. http://mindfulnessteachersuk.org.uk/pdf/MBCTImplementationResources.pdf

Lyubomirsky, S., King, L., & Diener, E. (2005). The benefits of frequent positive affect: does happiness lead to success?. Psychological bulletin,131(6), 803.

Lyubomirsky, S., Layous, K. (2013). How Do Simple Positive Activities Increase

Well-Being? Current Directions in Psychological Science, 22(1) 57–62.

Reibel, D. K., Greeson, J. M., Brainard, J. C., & Rosenzweig, S. (2001). Mindfulness-based stress reduction and health-related quality of life in a heterogeneous patient population. Gen Hosp Psychiatry, 23(4), 183-92.

Robinson, R., & Price, T. (1982). Post-stroke depressive disorders: a follow-up study of 103 patients. Stroke, 13, 635-641.

Shapiro, S. L., Schwartz, G. E., & Bonner, G. (1998). Effects of mindfulness-based stress reduction on medical and pre-medical students. Journal of Behavioral Medicine, 21, 581–599.

Shapiro, S. L., Carlson, L. E., Astin, J. A., & Freedman, B. (2006). Mechanisms of mindfulness. J Clinical Psych, 62(3), 373-386.

Shapiro, S. L., & Carlson, L. E. (2009). The art and science of mindfulness: Integrating mindfulness into psychology and the helping professions. Washington, DC, US: American Psychological Association.

Vago, D. R., & David, S. A. (2012). Self-awareness, self-regulation, and self-transcendence (S-ART): a framework for understanding the neurobiological mechanisms of mindfulness. Frontiers in human neuroscience, 6, 296.

Wagnild, G (1993). Development and psychometric evaluation of the resilience scale. Journal of Nursing Measurement, 1(2).

Williams, K. A., Kolar, M. M., Reger, B. E., & Pearson, J. C. (2001). Evaluation of a wellness-based mindfulness stress reduction intervention: A controlled trial. American Journal of Health Promotion, 15, 422–432.

Williams, J. M. G. (2010). Mindfulness and psychological process. Emotion, 10(1), 1.

**Appendix**

**Appendix A: Invitation email**

Hello,

As part of my Psychology Masters I am researching the effectiveness of mindfulness delivered via a phone app (Headspace). I am looking for volunteers to take part in the study which I hope to start next month.

Participants will be randomly allocated to either group A or group B. Group A will be asked to listen to Headspace for ideally 10 minutes a day (or every other day) on the tube, at home, or anywhere else for 30 days, and complete a 10-minute questionnaire at the start of the study, and following 10 and 30 days.

Group B will be asked to carry on with their everyday lives as usual, not listening to Headspace, but completing a questionnaire at the start of the study, and again following 10 and 30 days. When group B have completed the final questionnaire at day 30, they will have free access to Headspace for the following 30 days.

All participants will get free access codes to Headspace which can be downloaded on your smartphone. Questionnaires would be sent via email.

I appreciate life is busy and this might not be for you, but if you are interested or know someone who might be, then please email me back with the best email address to contact you/them on.

Thanks,

Louise

**Appendix B: Briefing email (mindfulness group)**

Title of study: Can an app enhance well-being?

Thank you for volunteering to take part in this research study. To participate in the study, you must be 18 years or over. You will need an iPhone/Android Smartphone to download the Mindfulness Headspace application. The study requires you to commit to self-administering around 10-20 minutes of Headspace ideally each day for the next 30 days and complete questionnaires at three timepoints. This study requires you to check your emails - reminders will be sent and you will be advised to follow a link and complete an online questionnaire at before starting to use the app, and again after 10 days and 30 days. The questionnaires are a satisfaction with life scale, perceived stress and resilience scales. You will also be asked to complete a short activity scale. These questionnaires will take no longer than approximately 15 minutes on each occasion.

Mindfulness is about the practice of the here and now and this research study will explore the effects a mindfulness smartphone app can have on wellbeing. In more detail, you will begin by responding to some questionnaires. You will then receive an access code and instructions on how to download the mindfulness app. You will be asked to spend the next 30 days listening to the app for approximately 10-20 minutes a day. You will then be asked to complete questionnaires as noted following 10 days and a final set at 30 days. There are no right or wrong answers so please answer ALL questions as truthfully as possible. All data is confidential and anonymous. If at any point during your participation you experience any psychological discomfort, please contact Headspace at science@headspace.com who will advise you further. You are volunteering to take part in the research and have the right to withdraw. If you wish to do so, please email me using your unique ID code provided to you by Survey Monkey; your data will then be destroyed.

All data will be protected in accordance with the Data Protection Act (1981) and kept securely by the researcher within a password protected area, only accessible to the Research Supervisor and University Examiners. The data will be securely maintained for five years and then destroyed. In the event of the publication of the study, data that is published will be generalised and anonymous. The full app for this study is usually chargeable by monthly subscription but full access to it will be free and fully available for the study period only. The app is created separately to this study and is not associated with the current research. This study has been approved by the London Metropolitan University Psychology Ethics Committee. Thank you for your time and participating in the study. Any questions, please contact me on loc0181@my.londonmet.ac.uk.

**Appendix C: Briefing email (wait-list group)**

Title of study: Can an app enhance well-being?

Thank you for volunteering to take part in this research study. To participate in the study, you must be 18 years or over. This study is looking at mindfulness, which is about the practice of the here and now. This research will explore the effects of a mindfulness smartphone app on well-being. As part of this study you have been randomly assigned to a particular research group, a vital role of the study. Consequently, this study requires you to carry on with your everyday activities and to check your emails, as reminders will be sent for you to follow a link and complete an online questionnaire within the next 48 hours, and then again following 10 days and finally 30 days. The questionnaires are a satisfaction with life scale, perceived stress and resilience scales. These questionnaires will take no longer than approximately 15 minutes on each occasion. Once again, you are a vital part of this study so please answer ALL questions within the questionnaires as truthfully as possible.

After 30 days from the start of the study, and after completion of the final questionnaires, you will be given free access to the full mindfulness programme (this is usually chargeable on a monthly subscription) for you to utilise as much as you want for the following 30 days. If, as a result of using the mindfulness practice you experience any psychological distress or wish to raise any queries as a result of the practice, please contact Headspace at science@headspace.com You are volunteering to take part in the research and have the right to withdraw anytime. If you wish to do so, please contact me quoting your unique ID code supplied by Survey Monkey; your data will then be destroyed.

All data will be protected in accordance with the Data Protection Act (1981) and kept securely by the researcher within a password protected area, only accessible to the Research Supervisor and University Examiners. The data will be securely maintained for five years and then destroyed. In the event of the publication of the study, data that is published will be generalised and anonymous. The full app for this study is usually chargeable by monthly subscription but full access to it will be free and fully available for the study period only. The app is created separately to this study and is not associated with the current research. This study has been approved by the London Metropolitan University Psychology Ethics Committee. Thank you for your time and participating in the study. Any questions, please contact me on loc0181@my.londonmet.ac.uk.
